# Supplementary material for: Splice-Junction-Based Mapping of Alternative Isoforms in the Human Proteome
Source: Cell Rep. Author manuscript; Available in PMC 2020 Jan 15. (PMC6961840; doi:10.1016/j.celrep.2019.11.026)
Supplement: 2 [file NIHMS1546469-supplement-2.zip › DF1/PXD006675/LeftAtrium/LeftAtrium_19_DPP7_LPGALLPAASGPLQLR.pdf]

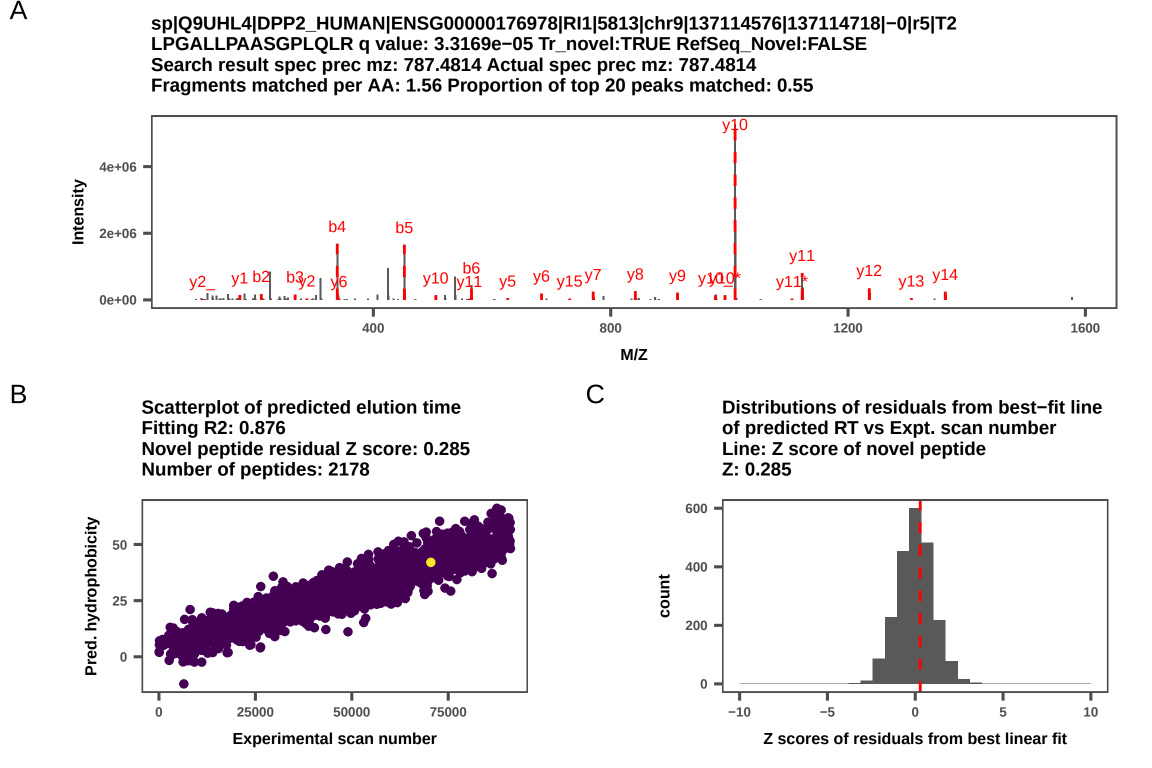
A
sp|Q9UHL4|DPP2_HUMAN|ENSG00000176978|RI1|5813|chr9|137114576|137114718|−0|r5|T2
LPGALLPAASGPLQLR q value: 3.3169e−05 Tr_novel:TRUE RefSeq_Novel:FALSE
Search result spec prec mz: 787.4814 Actual spec prec mz: 787.4814
Fragments matched per AA: 1.56 Proportion of top 20 peaks matched: 0.55
Intensity
4e+06
2e+06
0e+00
y10
b4
b5
y11
b6
y12
y14
y13
M/Z
400
800
1200
1600
B
Scatterplot of predicted elution time
Fitting R2: 0.876
Novel peptide residual Z score: 0.285
Number of peptides: 2178
Pred. hydrophobicity
50
25
0
0
25000
50000
75000
Experimental scan number
C
Distributions of residuals from best−fit line
of predicted RT vs Expt. scan number
Line: Z score of novel peptide
Z: 0.285
count
600
400
200
0
−10
−5
0
5
10
Z scores of residuals from best linear fit
